# Chemoprevention of Experimental Periodontitis in Diabetic Rats with Silk Fibroin Nanoparticles Loaded with Resveratrol

**DOI:** 10.3390/antiox9010085

**Published:** 2020-01-19

**Authors:** Ana Giménez-Siurana, Francisco Gómez García, Ana Pagan Bernabeu, Antonio Abel Lozano-Pérez, Salvador D. Aznar-Cervantes, José Luis Cenis, Pía López-Jornet

**Affiliations:** 1 Department of Bucal Medicine, Faculty of Medicine, University of Murcia, Av. Marqués de los Vélez, 2 Floor. 3008, Murcia, Spain; anakastel@msn.com (A.G.-S.); fjgomez@um.es (F.G.G.); 2 Department of Biotechnology, Instituto Murciano de Investigación y Desarrollo Agrario y Alimentario (IMIDA), C/Mayor 30150 La Alberca, Murcia, Spain; anapagan@um.es (A.P.B.); abel@um.es (A.A.L.-P.); sdac1@um.es (S.D.A.-C.); josel.cenis@carm.es (J.L.C.)

**Keywords:** resveratrol, periodontitis, silk fibroin nanoparticles, inflammation, IL-6, IL-1β, TGF-1β, diabetes

## Abstract

Objective: the objective of the present work is to study the effectiveness of treatment with silk fibroin nanoparticles loaded with resveratrol in experimental periodontitis in a diabetic rat model. Introduction: Periodontitis is an inflammatory pathology highly related to other diseases, such as type II diabetes. Both diseases have a specific inflammatory condition, with Interleukin (IL)-6, IL-1β and Transforming Grow Factor (TGF)-1β being the most relevant proinflammatory factors. Silk fibroin (SF) nanoparticles loaded with resveratrol (Res-SFN) are a new alternative as a treatment. Methods: 40 diabetic Sprague Dawley male rats were used and periodontitis was induced by ligation. The animals were divided into 5 treatment groups, and 1 mL of treatment was administered once a day for 4 weeks. The groups were: I: Carboxymethyl cellulose (CMC) 0.8%, II: CMC 0.8% + SF 1%, III: CMC 0.8% + RES-SFN 3 mg/mL, IV: CMC 0.8% + SF 1% + RES-SFN 3 mg/mL, V: Water. A peripheral blood sample was taken every week to quantify the inflammatory profile by ELISA (IL-6, IL-1β and TGF-1β). After 4 weeks the sacrifice was carried out and biopsies of the gum were taken. Results: Treatment with SF and RES-SFN reduced the amount of chemical inflammation mediators (with the exception of IL-1β in comparisons I-IV and II-IV (*p* > 0.05)), as well as the anatomopathological variables linked to it, in a significant way (*p* < 0.05). Conclusion: treatment with RES-SFN has reduced local inflammation in this experimental periodontitis model.

## 1. Introduction

Periodontal disease is a pathology of high prevalence in the population and presents a non-specific inflammatory condition. It is a disease closely related to systemic conditions, both inflammatory and non-inflammatory, such as diabetes or coronary heart disease [1]. During the inflammatory process that takes place in periodontitis, a significant increasing of the expression of interleukins, such as IL-1β, IL-6 or transforming growth factor (TGF-1β), which plays a fundamental role, occurs [2]. The levels of this transforming growth factor are also significantly incremented in diabetic patients [3,4].

IL-6, produced by most cells of the immune system, can have anti-inflammatory or pro-inflammatory effects depending on the circumstances in which its secretion is stimulated. Its anti-inflammatory effect is mediated by the inhibition of tumor necrosis factor α (TNFα) and IL-1 and the activation of the agonist receptor of IL-1 and IL-10. But, on the other hand, it is the mediator of induction of acute phase proteins, such as C-reactive protein, in addition to modifying body temperature when necessary. It is also responsible for the differentiation and proliferation of B lymphocytes, for the differentiation of monocytes in macrophages and for the induction of osteoclast formation, among other functions [5,6].

However, one of the most important roles of this interleukin is the regulation of bone metabolism [7]. Many studies support that this interleukin is involved in the development of a wide variety of diseases, and its presence and characterization can give us deeper details about the pathological anatomy situation in which the sample is found.

Finally, TGF-1β is a multifunctional cytokine that participates in angiogenesis, in the suppression of the immune system, in the synthesis of the extracellular matrix, apoptosis and in the inhibition of cell growth. Like IL-6, this cytokine has pleiotropic effects, both inflammatory and anti-inflammatory. It is responsible for stimulating inflammation because it turns out to be a chemotactic agent for both neutrophils, monocytes and lymphocytes and is also responsible for stimulating the production of other proinflammatory cytokines, such as IL-1 and IL-6 [8,9]. On the contrary, as an anti-inflammatory cytokine it works as a suppressor of the humoral response. Its secretion is carried out by lymphocytes, monocytes, platelets and neutrophils, and is one of the most relevant molecules in wound healing, tissue remodeling and regeneration [10]. Many studies have shown that levels in samples with gingivitis, chronic periodontitis and periimplantitis—of this mediator appear high, but have not been studied in depth in other oral diseases [11].

For a few years now, resveratrol (3,5,4′-trihydroxystilbene, RES) has been extensively studied and is well known for its antioxidant and anti-inflammatory abilities. It is a polyphenol extracted from the grape seed and other plants, which has various pharmacological activities, such as those mentioned above, in addition to vasoprotectors, anticancer and cardioprotective [12]. In addition, it has also been seen to have a protective effect on the loss and/or remodeling of alveolar bone in periodontitis and in the regulation of blood glucose levels in diabetic patients [13,14].

Silk fibroin itself has remarkable properties in regenerative medicine [15] and promoting wound healing [16], but, in addition, it has also been seen that in the form of nanoparticles, it has an excellent ability to store a drug inside, and serve as a vector for it [17,18,19,20]. The peptides that make up its structure have been shown to have a high therapeutic value both in the stimulation of glucose transport in adipocytes and in the stimulation of fibroblasts in wound healing (in both in vitro and in vivo studies), in addition to anti-inflammatory capacity, related to the decrease in levels of Cyclooxygenase-2 (COX-2), IL-6, and IL-1β among others [21].

The treatment by RES-SFN has been previously tested in other studies with inflammatory diseases with very attractive results, since it has been seen that encapsulation of the polyphenols in the nanoparticles can help to improve their capacities, improve their stability in the organism and prevent them from undergoing unwanted metabolic processes [17]. Additionally, it has also been demonstrated that the set of these two natural materials has no adverse effect on health or toxicity, regardless of the quantity and prolongation of its use [18], being a completely biocompatible treatment, and as soon as the tissue proteases digest the protein, the peptides are easily assimilated or reabsorbed by them. As mentioned at the beginning of the introduction periodontitis is an inflammatory pathology, and it has been found that many studies have tested some other kind of polyphenolic agents, but have encountered problems, such as those previously mentioned. With silk fibroin nanoparticles loaded with resveratrol it is wanted to overcome and solve these problems, in order to see the anti-inflammatory and healing applications of these natural chemicals in a disease that is very present in our society today. Periodontitis and diabetes are both diseases with a high prevalence in our society and they have an associative interrelation, so clinically it would be very positive to prevent them. Preventive treatment would play a very important role in diabetic patients that are in risk of suffering periodontitis.

The objective of this work has been to demonstrate if the treatment with silk fibroin nanoparticles loaded with resveratrol is effective or not in experimental periodontitis in a diabetic rat model.

## 2. Materials and Methods

### 2.1. Study Design and Ethical Considerations

#### 2.1.1. Animals

In order to carry out this study, after the approval of the Ethical Committee of Animal Experimentation of the University of Murcia 443/2018, 40 male Sprague Dawley rats of 2–3 weeks of age were used, weighing more than 150 g, fed ad libitum and maintained in cycles of 12:12 h light: darkness at 20 °C, for 4 weeks. The animals were acclimatized for 10 days before beginning the study.

#### 2.1.2. Experimental Design

##### Preparation and Evaluation of Treatments

Cocoons of Bombyx mori, obtained from silkworms reared in the sericulture facilities of the IMIDA (Murcia, Spain), were chopped in 5 pieces and boiled in 0.02 M Na_2_CO_3_ for 30 min to remove the glue-like sericin proteins. Then, raw fibroin was rinsed thoroughly with water and dried at room temperature for 3 days. The extracted fibroin was dissolved in 9.3 M LiBr (Acros Organics, Madrid Spain) for 3 h at 60 °C to generate a 20% w/v solution that was dialyzed against distilled water for 3 days (Snakeskin Dialysis Tubing 3.5 kDa MWCO, Thermo Scientific, Los Angeles, CA, USA) with eight total water exchanges. The resulting 7–8% w/v fibroin solution was stored at 4 °C until the preparation of the treatments. It was diluted at 1% w/v in the correspondent formulations. Silk fibroin nanoparticles loaded with resveratrol (RES-SFN) were prepared as it was previously described [18] and these nanoparticles were incorporated to the treatments at 3 mg/mL alone or in combination with SF solution at 1% w/v. RES-SFN were characterized prior use by spectrophotometry and dynamic light scattering in order to confirm the drug loading content of resveratrol and the hydrodynamic size and z-potential of the loaded nanoparticles, which were equivalent to the previously described for RES-SFN [18]. CMC 0.8% w/v was used as a carrier of the bioactive compounds (SF and/or RES-SFN) in all the treatments except the control group (water).

The designated experimental groups were: CMC 0.8% w/v (group I, considered as pla-cebo); CMC 0.8% w/v + SF 1% w/v (group II); CMC 0.8% w/v + RES-SFN 3 mg/mL (group III); CMC 0.8% w/v +SF 1% w/v + RES-SFN 3 mg/mL; water (group V, control). All of them were stored at 4 °C, protected from UV light prior to its use.

##### Induction of Diabetes

Initially diabetes was induced by an intraperitoneal dose 50 mg/kg of Estreptozocine (STZ) (Sigma Aldrich©, MerckGaA Darmstadt, Germany) dissolved in citrate buffer 0.09 M (Sigma Aldrich©, MerckGaA Darmstadt, Germany). Various checks were made with a glucometer to see the initial state (non-diabetic) and the evolution of diabetes, considering that the animal is diabetic when the glucose level is above 600 mg/dL.

##### Induction of Periodontitis and Treatment

Four days after the induction of diabetes, periodontitis was performed by introducing 4-0 silk thread around the lower incisors, following the protocol described by Ionel et al. [22]. The rats were randomly divided into the five experimental groups, previously described, and were administered orally, daily, 1 mL of corresponding treatment for 4 weeks.

The treatment groups are shown in Table 1:

To evaluate the inflammation using ELISA and pathological anatomy, the evaluator was blinded, without knowing at any time what samples belonged to each of the study groups.

##### Sample Processing

On the first day of the study, an initial intake of: weight, blood glucose and blood samples were obtained from each of the animals. This same process was repeated once a week for the next 4 weeks. After completing the study, the animals were sacrificed by means of a CO_2_ Chamber. 

##### Enzymatic Assay

In order to analyze the evolution of the markers of inflammation, the plasma was separated from the rest of the blood of the samples, by means of the centrifugation technique at 1500 rpm during 10’ at 4 °C. Once the plasma samples were separated, three ELISA tests were performed to determine the concentrations of the different interleukins studied:

IL-6, IL-1β Y TGF-β1, according to their respective manufacturer protocols: Rat IL-6 ELISA KIT of Diaclone^®^ (Besançon, France), Rat Transforming Growth Factor 1β (TGF- 1β) ELISA KIT of Cusabio^®^ (Houston (TX), USA), and Rat IL-1β (Interleukin 1 β) ELISA KIT of Elabscience^®^ (Houston (TX), USA). The CLARIOstarplus system and the MARS^®^ Software, both of BMG LABTECH© (Ortenberg, Germany), were used to read the plates.

##### Pathological Anatomy

After euthanasia, tissue gum samples were collected, which were fixed, included in paraffin, cut to 4 microns and stained with hematoxylin-eosin, Masson’s trichrome and Periodic Acid-Schiff (PAS) by the usual anatomopathological method. Three slides of each block were obtained. For morphometric analysis, the images were digitized using the Leica Biosistems^®^ Digital Image Hub online software (Wetzlar, Germany). The study variables were determined: thickness and structure of the epithelium, state of the basal membrane, quantitative measurement of the inflammatory area and the condensation of the connective tissue, according to the criteria described by authors such as Camacho-Alonso et al. [23], Bosca et al. [24] and Ionel et al. [25].

To quantify and evaluate the epithelium and the basal membrane, 5 measures of epithelium thickness (µm) were made for each of the samples. The quantification of inflammatory cells present in each of the tissue samples (gum) was also performed. In order to measure the area of inflammation, several sources of inflammation were chosen from each sample at an increase of ×10. To analyze the area of inflammation Leica QWinn Pro image analysis software V2.2^®^ (Wetzlar, Germany) was used. In each of the images obtained, the area occupied by all the proinflammatory cells present in the sample was quantified versus the total area of the image. The first thing that was done was to make sure that the measurement rule was well calibrated. The examinator A.G.S. did a calibration test, using 10 different images obtaining a 95,6% of reproducibility.

The area of each proinflammatory cell in each image was delimited and a sum was made to see what the total area of inflammatory cells was. Once the total area of inflammation was calculated, it was divided for the total area of the image. After this, the percentage of area of inflammation was calculated, representing the percentage of inflamed area with respect to the total area of the sample.

The results obtained in the ELISAs and histopathology measurements were analyzed using non-parametric U-Mann Whitney and W-Wilcoxon statistical tests with *p* < 0.05 for the treatment groups, with the SPSS software© version 12.0 (Chicago, IL, USA).

## 3. Results

### 3.1. Glycemic Index and Weight

During the three days of treatment with STZ, daily measurements of the glycemic index were obtained and it was found that all rats were diabetic. A comparison of the initial blood glucose was performed between all groups (*p* = 0.045) to check if all the rats were within the same blood glucose range. In addition, it was also found throughout the study that all rats were still diabetic (for all of them the glycemic index value exceeded 600 mg/dL). During the study, the animals suffered a continuous weight loss from the beginning to the end with significant differences (initial weight—final weight, *p* = 0.000).

### 3.2. Elisa Assay

**Figure 1:**
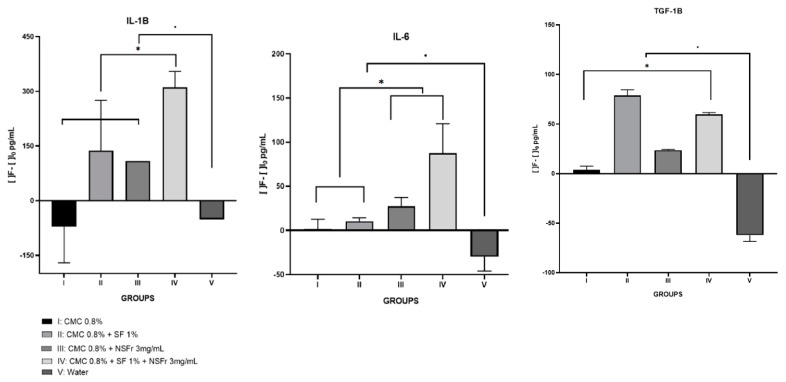
Graph describing the differences between the different interleukins quantified by ELISA method in the different groups. * means difference between groups of treatment. • means difference between the groups of treatment and the control.

Interleukin 6: there were significant differences between the beginning and the end of each of the fibroin and resveratrol treatment groups (*p* < 0.05). Among the treatment groups, the one that stands out the most is group IV (*p* = 0.021). Group II and group III follow the same trend as group IV, but with less intensity (Figure 1).

Interleukin 1β: with this IL we observe the same behavior as with the IL-6, the treatment groups present statistically significant differences (*p* < 0.05) with respect to the control, and it is the group IV that presents the greatest difference (*p* = 0.021). Group II and III have the same behavior, but to a lesser extent.

Transforming Growth Factor 1β: with this pro-inflammatory factor, we see that all the groups present statistically significant differences (*p* < 0.05), but in this case it is the group II that stands out (*p* = 0.021). Group III and IV, present the same behavior, but to a lesser extent.

All numerical data are expressed in Table 2.

### 3.3. Pathological Anatomy

**Figure 2:**
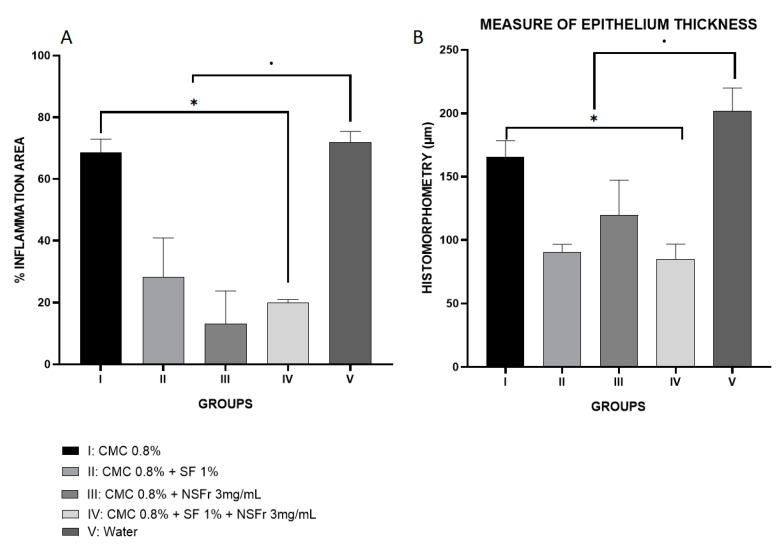
Graph **A**: % of area of inflammation by treatment groups. Graph **B**: representation of the depth measurement of the epithelium in micrometers (µm) by treatment groups. * means difference between groups of treatment. • means difference between the groups of treatment and the control.

With regard to histomorphometry, all treatment groups have statistically significant differences with respect to the control (*p* < 0.05). In the measurement of the epithelium, group IV stands out because it has the lowest thickness (*p* = 0.009), followed by group III and group II. The group with the lowest area of inflammatory focus is III (*p* = 0.001), although it presents little difference with group IV. Group II also shows significant differences, but to a lesser extent (Figure 2).

**Figure 3:**
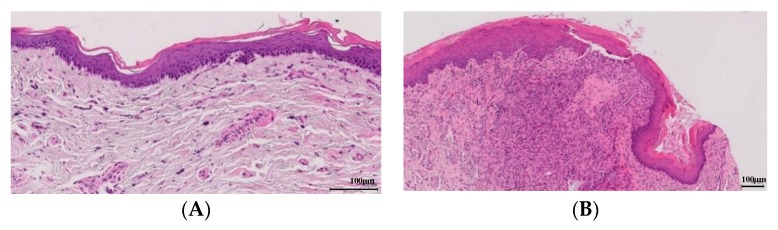

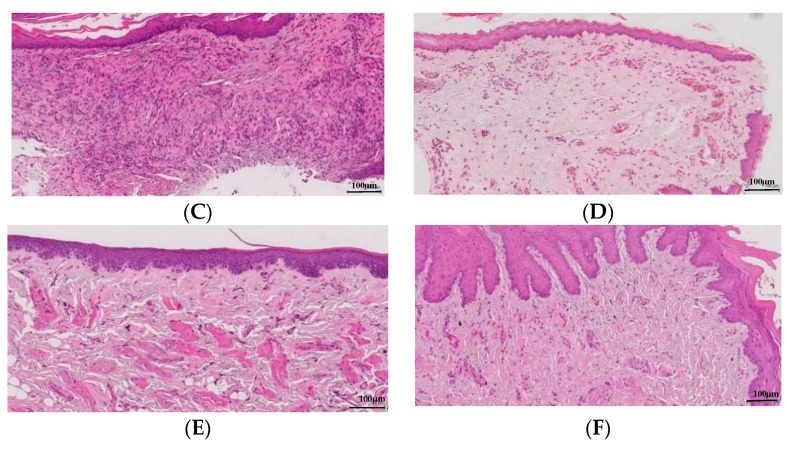
(**A**) (Edema and slight inflammation, group V), (**B**,**C**) (cellular granulation tissue slightly vascularized, group V), (**D**) (profuse vascularization and slight inflammation, group IV), (**E**) (edema and slight inflammation, group IV) and (**F**) (slight inflammation with macrophage appearance, group IV). It is only shown control group and the treatment that revealed the best results (CMC + SF + RES-SFN).

Pathological anatomy measurements show the lowest values in treatment groups II, III and IV, the latter being the most prominent among all of them (Figure 3). All groups have integrity of the epithelium membrane. Table 3 shows the average measurements of every anatomopathological feature.

## 4. Discussion

The treatment using RES-SFN shows the best overall results in terms of decreasing levels of proinflammatory interleukins due to periodontitis, in addition to presenting a smaller amount of pathological anatomy variables.

### 4.1. ELISA Assays

In group I there is no change in inflammatory levels since it acts only as a carrier vector for the rest of the treatments, both of the fibroin, of the fibroin nanoparticles with fibroin, and of the silk nanoparticles loaded with resveratrol. As Friz & Becker [26] described, CMC is an inert substance, without any activity that could affect the objective of this study.

The basal values of IL-1β are not close to 0 because this interleukin produces an inflammatory process that is related not only to local factors, but also to systemic factors, such as stress or the condition of chronic inflammation produced by diabetes

The IL-6, is the one that presents the most association with the development of the periodontitis due to its participation in bone remodeling, presents some insignificant initial levels, —since periodontitis was not developed in the initial blood sample, and we see that finally the values decrease significantly, remaining at values of 100 pg/mL or below this value. If we look at the results obtained with this IL, all the proposed treatments are effective in preventing the progress and development of periodontitis. However, if we deepen, we see that the one that stands out the most is treatment administered to group IV. These same results were also conclusive in the study by Ebersole et al. [27], where it was determined that if the periodontal disease does not develop, the levels of this interleukin end up decreasing, since the treatment does not allow the pathology to go further and move towards stages that they carry inflammation. Correa et al. [28] described similar results for both IL-6 and IL-1β: resveratrol helped decrease the values of these interleukins in experimental periodontitis, but our study goes further, the values that better prognosis present are those of group IV, those treated with RES-SFN. IL-1β is an interleukin involved in inflammation caused by bone remodeling, and as described by Hosokawa et al. [29], its expression is related to the one of IL-6, and as we see that in both cases the concentrations at the end of study decreasing with respect to the initial values, we can affirm that periodontal disease has not continued to develop. As with IL-6, it is again the group IV treatment that has the best evolution throughout the study and that helps to reduce the level of proinflammatory cytokines present to a greater extent. As described by Gürkan et al. [30], TGF-1β is a cytokine associated with periodontal disease, so we see levels at the beginning of treatment so low and irrelevant, as is the case with IL-6. Skaleric et al. [11] showed that the levels of this inflammatory factor were increased if periodontitis developed, so the values, both initial and final, close to 0 suggest that the treatment has not allowed the disease to develop.

### 4.2. Pathological Anatomy

The study of pathological anatomy helps us to reaffirm the results obtained with the ELISA essays. 

As expected, group V has a greater area of inflammation. On the other hand, groups II, III and IV show this much lower value, that is, the treatment shows that the disease has not advanced. In this case, the group that stands out the most is III, but the differences with group IV are minimal. This small difference may be due to the fact that the technique used in this section is less accurate than the ELISA one, in a way, that we can say that the group that stands out among all is still group IV.

When there is inflammation present in the organism, as occurs in the gum with the development of periodontitis, the cells of the basal membrane of the epithelium migrate to deeper areas leading to the appearance of papilla, so, the greater the inflammation, greater thickness of the epithelium. In this way, the results obtained, indicate that in the groups of treatment with silk fibroin and resveratrol correspond to the measures of smaller thickness of the epithelium, being the group IV the one that offers the best results.

Lorencini et al. [31] demonstrated that greater collagen compaction corresponds to a greater presence of inflammation in the sample, and in our case, it would correspond to an advance in periodontitis. Séguier et al. [32] correlated the condensation of collagen with the presence of proinflammatory factors, seeing that the higher the concentration of these, the greater the compaction of the collagen. According to our results, we observe that treatment groups II, III and IV have a level 1 collagen compaction, which would correspond to little inflammation, so we can deduce that the treatment has not allowed periodontitis to progress.

In addition, it has been seen in some studies [33] that angiogenesis is related to the development of periodontitis, so that a greater or lesser presence of vessels in the samples will suggest the progression or not of the disease throughout the treatment. Treatment groups II, III and IV, showing such a low level of vessel formation, show again that they are suitable treatments for the prevention of periodontitis.

### 4.3. Silk Fibroin and Resveratrol

Correa et al. [28] and Lozano et al. [17], among many others, have already described the anti-inflammatory properties of the treatments themselves, both of resveratrol and of silk fibroin, and which are capable of reducing inflammation in a wide spectrum of diseases, such as diabetes, irritable bowel syndrome or arthritis. In addition, the high antioxidant potential has also been demonstrated with in vitro studies.

On the other hand, as authors such as Zhao et al. [34] and Melke et al. [35] state, silk fibroin does not leave us indifferent as to its potential as a biomaterial. It has great diversity in terms of design: from tights, to sponges and gels. But the fibroin design that best suits our study is the structure in the form of nanoparticles, since its external structure can be modified to modify its absorption, specific recognition, or electrostatic interactions with other molecules and surfaces, which is conditioning to determine its potential. In addition, they have a high potential for the administration of therapies and drugs in a selective and controlled manner, gene therapy, cancer diagnosis, protein administration or administration of antitumor agents [36]. In addition, it has also been shown that silk fibroin nanoparticles are a good method for administering substances in a specific way, since the problems that many authors have had regarding the toxicity of nanoparticles are resolved, as is the case with nanoparticles of a metallic nature [37,38] and with respect to the bioavailability of the agents contained therein [39].

But the novelty of the current study is the use of RES-SFN, we can see that with this treatment the best results are obtained, both for the decrease in the concentration of proinflammatory interleukins, and for the results of pathological anatomy. We believe, then, that the silk nanoparticles used in this study are a good method to load and release resveratrol in the area of interest, gum and periodontal tissue. The nanoparticles have a structure and properties that are suitable for adhering to the gingival tissue, being able to release their contents gradually, and one in turn degrading without causing any harmful effect to the organism and leaving as a single residue a series of peptides easily assimilated by the body.

Rats have been a perfect animal experimental model for this study: it has been possible to demonstrate the high potential of this treatment based on natural polyphenols and silk fibroin. Some studies demonstrate that poverty can be a risk factor for both diabetes and periodontitis, due to poor oral hygiene and unhealthy and poor diet that the lowest strata of society can suffer [40,41], so after this, perhaps a study with diabetic humans suffering from periodontitis, since the treatment does not generate neither toxicity nor associated problems, shows us even more satisfactory results and we can consider this treatment as a new therapy to combat progress and aggressiveness of a disease as globally widespread as periodontitis. Apart of this, it also has to be considered that human periodontitis is a chronic inflammation caused by an opportunistic infection and in this study, it has been created an acute inflammation by ligature. This study, unfortunately, does not allow an extrapolation of interspecies results and these same results cannot be ensured in humans with this treatment, although resveratrol and fibroin have been tested separately, obtaining very good results.

In spite of everything, we must also mention that a non-parametric statistical analysis had to be performed due to the small size of the population sample (*n* = 40), with 8 rats per group not being sufficient to be able to perform parametric significance tests.

## 5. Conclusions

The results of this study let us think that treatment with RES-SFN is the one that has obtained the best results for the treatment of periodontal disease in this model. Liquid silk fibroin along with the RES-SFN treatment revealed better results than these treatments separately, so this positive synergy could also be considered as a preventive treatment for periodontitis.

## Figures and Tables

**Table 1:** Groups of treatment completely described.

Groups	Treatment
I	CMC 0.8% (placebo)
II	CMC 0.8% + SF 1%
III	CMC 0.8% + RES-SFN 3 mg/mL
IV	CMC 0.8% +SF 1% + RES-SFN 3 mg/mL
V	Water (Control)

**Table 2:** Initial description of the means of the results obtained from the ELISA tests before and after treatment, initial weight and blood glucose level, and the difference between the final and initial interleukin values. Group I: Carboxymethyl cellulose (CMC) 0.8% (placebo), group II: CMC 0.8% + SF 1%, group III CMC: 0.8% + RES-SFN 3 mg/mL, group IV: CMC 0.8% +SF 1% + RES-SFN 3 mg/mL, group V: water (Control).

Variable	Group. *Median (IR)*					*p-Value*
	I	II	III	IV	V	
INITIAL IL-1β (pg/mL)	1421.27 (1349.31–1446.21)	1378.71 (1312.11–1448.89)	1486.99 (1468.77–1504.61)	1698.71 (1680.86–1718)	1056.57 (944.07–1159.07)	
FINAL IL1β (pg/mL)	1434.31 (1222.17–1544.79)	1199.67 (1106.75–1432.35)	1390.8 (1348.54–1492.23)	1402.46 (1326.93–1430.5)	1646.15 (1563–1737.05)	
INITIAL IL-6 (pg/mL)	100.33 (78.97–132.51)	47.09 (39.44–57.25)	67.1 (55.22–75.85)	181.37 (153.56–204.6)	103.66 (96.47–118.35)	
FINAL IL-6 (pg/mL)	100.99 (82.55–119.97)	39.14 (30.19–44.67)	43.26 (17.99–53.97)	93.14 (81.58–97.41)	136.89 (131.47–146.26)	
INITIAL TGF-1β (pg/mL)	4.69 (2.21–7.86)	78.72 (75.88–84.48)	80.26 (77.99–81.19)	62.12 (59.53–63.03)	43.89 (41.45–50.73)	
FINAL TGF-1β (pg/mL)	0.81 (0–3.24)	0 (0–0)	56.13 (55.56–56.7)	0.6 (0–2.4)	106.02 (101.98–109.82)	
INITIAL WEIGHT (g)	330 (322.5–332.5)	360 (349–390)	321.5 (318–347)	325 (294–390)	351 (304–374.5)	0.303
INITIAL GLUCEMIA (mg/dL)	591.5 (481.5–600)	591 (583–600)	525.5 (495–593.5)	569.5 (491–589)	502 (471–514)	0.045
IL-1βDIFF (pg/mL)	−71.25 (−170.54–199.11)	137.26 (−53.63–275.54)	108.87 (−23.45–156.07)	311.25 (286.79–354.64)	−510.3 (−792.98–403.93)	0.014
IL-6 DIFF (pg/mL)	1.58 (−3.58–12.55)	10.5 (7.53–14.3)	27.59 (21.88–37.23)	87.6 (58.44–120.73)	−29.79 (−46.04–16.88)	0.002
TGF-1β DIFF (pg/mL)	3.88 (−0.7–7.52)	78.72 (75.88–84.48)	23.57 (22.43–24.49)	59.79 (58.63–61.52)	−62.13 (−68.37–51.25)	0.001

**Table 3:** Pathological anatomy measures of each of the biopsies of the treatment groups. Formation of vessels: 0 nothing, 1 little, 2 moderate, 3 abundant. Collagen compaction: 0 very lax, 1 lax, 2 compact, 3 very compact. Composition of the basement membrane: 1 integral, 2 not integral.

Measurement	Group, *Median (IR)*				
	I	II	III	IV	V
Inflammation area (x̄ %)	68.734	28.250	13.108	19.805	71.978
Epithelium thickness (x̄, µm)	165.560	90.255	119.545	85.860	189.297
Vessel formation	2	1	1	1	3
Collagen	2	2	1	0	3
Membrane integrity	1	1	1	1	1
